# Special Issue of the Manufacturing Engineering Society 2019 (SIMES-2019)

**DOI:** 10.3390/ma13092133

**Published:** 2020-05-05

**Authors:** Eva María Rubio, Ana María Camacho

**Affiliations:** Department of Manufacturing Engineering, Industrial Engineering School, Universidad Nacional de Educación a Distancia (UNED), St/Juan del Rosal 12, E28040 Madrid, Spain; amcamacho@ind.uned.es

**Keywords:** additive manufacturing, 3D printing, forming, machining, metrology, industry 4.0, green manufacturing, modeling and simulation, quality in manufacturing, technological and industrial heritage

## Abstract

The Special Issue of the Manufacturing Engineering Society 2019 (SIMES-2019) has been launched as a joint issue of the journals “Materials” and “Applied Sciences”. The 29 contributions published in this Special Issue of Materials present cutting-edge advances in the field of manufacturing engineering focusing on additive manufacturing and 3D printing, advances and innovations in manufacturing processes, sustainable and green manufacturing, manufacturing of new materials, metrology and quality in manufacturing, industry 4.0, design, modeling, and simulation in manufacturing engineering and manufacturing engineering and society. Among them, these contributions highlight that the topic “additive manufacturing and 3D printing” has collected a large number of contributions in this journal because its huge potential has attracted the attention of numerous researchers over the last years.

After the complete success of the first edition [1] with 48 contributions on emerging methods and technologies, the Special Issue of the Manufacturing Engineering Society 2019 (SIMES-2019) [2] was launched as a joint issue of the journals “Materials” and “Applied Sciences”.

Once again, this Special Issue was promoted by the Manufacturing Engineering Society (MES) [3] of Spain, with the aim of covering the wide range of research lines developed by the members and collaborators of the MES and other researchers within the field of manufacturing engineering.

In this Special Issue of the journal Materials, 29 contributions to cutting-edge advances in different fields of the manufacturing engineering have been collected. In particular, in additive manufacturing and 3D printing [4,5,6,7,8,9,10,11]; sustainable and green manufacturing [12,13,14,15,16]; metrology and quality in manufacturing [17,18,19,20,21]; advances and innovations in manufacturing processes [22,23,24,25]; manufacturing of new materials [26,27,28]; design, modeling, and simulation in manufacturing engineering [29,30] industry 4.0 [31] and manufacturing engineering and society [32].

Among all of them, the topic additive manufacturing and 3D printing stands out for the number of contributions it has had in this Special Issue showing the interest that this topic arouses, currently, among researchers, the industry and the public in general. The works focus on the manufacturing processes [4,5], the characterization of materials and parts [6,7,8], the estimation of times [9,10] and the dimensional and geometrical quality of the obtained pieces [11]. The next topics by the number of contributions are sustainable and green manufacturing and metrology and quality in manufacturing with five contributions each. The first one gathers four works about new sustainable lubrication/cooling techniques used in removal processes [12,13,14,15] and the other about reusing waste in ecological cement [16]. The second one collects research about the optimization of laser tracker location on verification process [17], estimation of an upper bound to the value of the step potentials from grounding resistance measurements [18], enhanced positioning algorithm by mesh elements, recalculation and angle error orientation [19], industrial calibration procedure for confocal microscopes [20] and, finally, the development and validation of a calibration gauge for length measurement systems [21]. The Special Issue also collects four pieces of work in the topic advances and innovations in manufacturing processes. In particular, an experimental and numerical analysis of the compression of bimetallic cylinders [22], a study about thermal analysis and Raman spectroscopy in composite-forming processes [23], a new deformation-assisted joining of sheets to tubes by annular sheet squeezing [24] and an analysis of the influence of the rotary dresser geometry on wear evolution and on the grinding process [25]. In addition, the Special Issue shows three papers in the topic manufacturing of new materials. Concretely, two about thermoplastic carbon fiber composites C/TPU [26,27] and, other, about an Al-SiC metal matrix composite [28]. It counts also with two works in design, modeling, and simulation in manufacturing engineering about the indentation process [29] and the cold expansion process [30]; one, in the topic industry 4.0 about the application of the supply chain to shipbuilding [31] and the other in the topic manufacturing engineering and society about a new risk methodology based on control charts to assess occupational risks in manufacturing processes [32].

Finally, it remains to highlight that in just four months since the publication of the first work [18], all the papers present prominent activity in their “article metrics”; being remarkable that some of the papers, belonging to this Special Issue, have already had more than five hundred abstract and full-text views, which is clear evidence of the interest readers have for all these topics.
